# Graphene Oxide–Silver Nanoparticle Nanocomposites Induce Oxidative Stress and Aberrant Methylation in Caprine Fetal Fibroblast Cells

**DOI:** 10.3390/cells10030682

**Published:** 2021-03-19

**Authors:** Yu-Guo Yuan, He-Qing Cai, Jia-Lin Wang, Ayman Mesalam, Abu Musa Md Talimur Reza, Ling Li, Li Chen, Chen Qian

**Affiliations:** 1 College of Veterinary Medicine/Joint International Research Laboratory of Agriculture and Agri-Product Safety, the Ministry of Education of China, Yangzhou University, Yangzhou 225009, China; chq1071357042@163.com (H.-Q.C.); wjl15861382137@163.com (J.-L.W.); ll13665200796@163.com (L.L.); 007238@yzu.edu.cn (L.C.); 13905271422@163.com (C.Q.); 2 Jiangsu Co-Innovation Center for Prevention and Control of Important Animal Infectious Diseases and Zoonoses/Jiangsu Key Laboratory of Zoonosis, Yangzhou 225009, China; 3 Department of Theriogenology, Faculty of Veterinary Medicine, Zagazig University, Zagazig 44519, Egypt; aymanmesalam@gmail.com; 4 Institute of Biochemistry and Biophysics, Polish Academy of Sciences, Pawińskiego 5a, 02-106 Warsaw, Poland; talimurku@gmail.com

**Keywords:** graphene oxide, silver nanoparticles, caprine fetal fibroblast cells (CFFCs), reactive oxygen species (ROS), epigenetic

## Abstract

Graphene oxide–silver nanoparticle (GO-AgNPs) nanocomposites have drawn much attention for their potential in biomedical uses. However, the potential toxicity of GO-AgNPs in animals and humans remains unknown, particularly in the developing fetus. Here, we reported the GO-AgNP-mediated cytotoxicity and epigenetic alteration status in caprine fetal fibroblast cells (CFFCs). In brief, the proliferation and apoptosis rate of GO-AgNP-treated CFFCs (4 and 8 µg/mL of GO-AgNPs) were measured using the cell-counting kit (CCK-8) assay and the annexin V/propidium iodide (PI) assay, respectively. In addition, the oxidative stress induced by GO-AgNPs and detailed mechanisms were studied by evaluating the generation of reactive oxygen species (ROS), superoxide dismutase (SOD), lactate dehydrogenase (LDH), malondialdehyde (MDA), and caspase-3 and abnormal methylation. The expression of pro- and anti-apoptotic genes and DNA methyltransferases was measured using reverse transcription followed by RT-qPCR. Our data indicated that GO-AgNPs cause cytotoxicity in a dose-dependent manner. GO-AgNPs induced significant cytotoxicity by the loss of cell viability, production of ROS, increasing leakage of LDH and level of MDA, increasing expression of pro-apoptotic genes, and decreasing expression of anti-apoptotic genes. GO-AgNPs incited DNA hypomethylation and the decreased expression of *DNMT3A*. Taken together, this study showed that GO-AgNPs increase the generation of ROS and cause apoptosis and DNA hypomethylation in CFFCs. Therefore, the potential applications of GO-AgNPs in biomedicine should be re-evaluated.

## 1. Introduction

Nanotoxicity involves the understanding of the adverse biological effects of nanoparticles using both in vitro and in vivo model systems such as cells, tissues, organs, and organisms [1]. In vitro assays are the first approach to determining the cytotoxicity of nanomaterials. Several studies have been dedicated to examining the effects of graphene and graphene-related nanomaterials in various cell culture systems, including HeLa, MCF-7, SKBR3, NIH3T3, epithelial lung carcinoma, primary mouse embryonic fibroblast, human breast cancer, and ovarian cancer cells [2,3,4,5]. It has been shown that graphene and graphene-related nanomaterials lead to a pro-inflammatory response in the liver and kidney [6,7], cause genotoxicity and DNA damage [8], and adversely impact the function of the lungs, heart, intestines, and spleen [9,10,11]. In addition, depending on the size, oxidative status, and concentration of nanoparticles, and the used experimental model, graphene, graphene oxide (GO), and reduced GO have shown different levels of cytotoxicity [12,13]. Previous research showed the involvement of different nanocomposites in the abnormal methylation of mammalian DNA [14]. However, the role of the graphene-derived nanocomposite in the aberrant methylation status of DNA in livestock species has not been reported yet.

Silver nanoparticles (AgNPs) are one of the most frequently used nanoparticles in a variety of biomedical applications [15]. Recently, investigating the potential of using hybrid nanomaterials such as graphene oxide–silver nanoparticle (GO-AgNPs) nanocomposites is becoming popular in biomedical research because of their unique functions and properties [16]. For example, attaching AgNPs on to the surface of GO sheets can prevent the AgNPs from aggregating, allowing for a more controlled release of AgNPs+ ions, and lead to an increase in antibacterial and anticancer activity [17,18]. The commercial use of this kind of nanocomposite might cause a slow but chronic exposure to human, animal, and other forms of environmental elements. For instance, exposure to AgNPs causes their accumulation in cells and animal tissues (such as the heart, kidney, and other organs) [19,20,21], which may interfere with transport pathways, nuclear signaling, endocytosis, reproductive behavior, and general defenses by alteration of gene expression [22,23]. Therefore, the potential toxicity of our synthesized GO-AgNPs needs to be explored in detail.

Our previous study aimed at the use of nanomaterials in veterinary treatment showed that 1 µg/mL of AgNPs is effective against multidrug-resistant (MDR) bacteria in dairy goats [24]. However, AgNPs could be translocated to the bloodstream and transported throughout the organs of the body, including reproductive organs, which might cause disruption of reproductive system development, birth weight reduction, and other fetal–maternal disorders [25]. Thus, it becomes essential to investigate the potential cytotoxic and genotoxic effects of our newly synthesized GO-AgNP nanocomposite before its application in the treatment of bovine and caprine mastitis. The current research was undertaken to investigate the cytotoxic and genotoxic effects of GO-AgNPs in caprine fetal fibroblast cells (CFFCs), which is a suitable in vitro model for studying the nanomaterial-mediated toxicity to the fetus. The results showed that GO-AgNPs cause aberrant methylation of CFFCs. To the best of our knowledge, this is the first evidence showing that GO-AgNPs could impair the epigenetic status of fetal fibroblasts in livestock species.

## 2. Materials and Methods

### 2.1. Chemicals

All chemicals and reagents were purchased from Sigma-Aldrich (St. Louis, MO, USA) unless otherwise stated.

### 2.2. Synthesis and Characterization of GO-AgNPs

A GO-AgNP nanocomposite was synthesized using the biomolecule quercetin, as described previously [26], and then lyophilized and kept in a lab at 4 °C until use. Briefly, 50 mg of GO was dispersed in 30 mL of water and sonicated for 60 min. Then, 1 mM AgNO_3_ was dissolved in 15 mL of water in a 500 mL round-bottom flask. Next, 30 mL of the GO dispersion was added, followed by addition of 5 mL of aqueous 1 mM quercetin, and then stirred at 60 °C for 12 h. The resultant mixture was washed and centrifuged three times with water. Physicochemical characterization of GO-AgNPs was checked by Fourier-transform infrared spectroscopy and X-ray diffraction. The size and shape were observed under a transmission electron microscope (TEM; HT7800, Hitachi High-Technologies Corporation, Tokyo, Japan).

### 2.3. Cell Culture

CFFCs were isolated from 70-day-old fetuses that were recovered surgically from a Boer goat obtained from the Yangzhou University farm, as previously described [27]. Briefly, pregnant goats were anesthetized using an intramuscular injection of serazine hydrochloride (0.02 mL/kg body weight), and fetuses were collected. After removal of the head and internal organs, the remaining tissues of fetuses were dissociated into small pieces using scissors and digested with 0.25% trypsin (Thermo Fisher Scientific, Waltham, MA, USA). Then, cells were washed three times, centrifuged to recover them, and cultured in Dulbecco’s Modified Eagle’s Medium/F12 (DMEM/F12; Thermo Fisher Scientific, Waltham, MA, USA) supplemented with 10% fetal bovine serum (FBS; Hangzhou Sijiqing Hangzhou, China) at 37 °C in a humidified atmosphere of 5% CO_2_. The cells were used at passages 3–10.

### 2.4. Cell Viability Assay

The cell viability was assessed by using an in vitro cell-counting kit (CCK-8; Rockville, MD, USA) assay, as described previously [28]. CFFCs were seeded in a 96-well or 6-well plate and cultured for 24 h to allow adherence and stabilization. GO-AgNPs were sonicated for 20 min before use. Then, the GO-AgNP suspension was dispersed in DMEM/F12 at different concentrations (1, 4, 8, 12, and 16 μg/mL) for 24 h at 37 °C. After culture, 10 μL of CCK-8 was added to each well and incubated for 30 min at 37 °C in the dark. The absorbance at 450 nm was measured using a microplate reader (BioTek Synergy 2, Winooski, VT, USA). After calculating the LC50 value from the resultant cell viability data, 4 and 8 μg/mL concentrations were selected for further experiments. This study was designed and blinded throughout all stages of the methodological process.

### 2.5. Cell Morphology

CFFCs were seeded in a 24-well plate for 24 h and then treated with 0, 4, and 8 μg/mL of GO-AgNPs for 24 h. Cell morphology was observed using an Olympus BX-UCB microscope (Tokyo, Japan).

### 2.6. Annexin V–FITC/PI Staining Assay

CFFCs were seeded in a 75 mm culture plate and treated with different concentrations of GO-AgNPs (0, 4, and 8 μg/mL) for 24 h. Cell apoptosis of CFFCs was detected by the annexin V–FITC and propidium iodide (PI) staining assay according to the manufacturer’s instructions (Bipec Biopharma Corporation, Warminster, PA, USA). The cells were harvested, centrifuged for 5 min, rinsed with phosphate-buffered saline (PBS) twice, resuspended in 500 μL of binding buffer containing 5 μL of PI and 5 μL of annexin V–FITC, and then incubated for 15 min at room temperature in the dark. The cell suspension was analyzed by flow cytometry to analyze the apoptotic rate.

### 2.7. Measurement of ROS Production

Dichlorodihydrofluorescein diacetate (DCFH-DA) was used to detect intracellular ROS induced by 0, 4, and 8 μg/mL of GO-AgNPs, as described earlier [28]. In brief, CFFCs were incubated in 10 μM DCFH-DA for 30 min at 37 °C. The cells were rinsed with PBS twice, and then the intracellular accumulation of ROS was measured by flow cytometry (Beckman-Coulter, Irving, TX, USA).

### 2.8. Measurement of Total Superoxide Dismutase (SOD) Enzyme Activity

The SOD assay kit (Beijing Solarbio Science & Technology, Beijing, China) was used to detect the activity of SOD in the CFFCs treated with 0, 4, and 8 μg/mL of GO-AgNPs for 24 h [28]. In brief, after treatment with GO-AgNPs, the cells were washed with PBS twice and lysed with lysis buffer on ice. The lysates were then centrifuged for 15 min. Then, the supernatant was analyzed with a UV–VIS spectrophotometer (Nanodrop, Thermo, Waltham, MA, USA) at 550 nm.

### 2.9. Measurement of Malondialdehyde (MDA) Production

MDA, a convenient index for detecting the extent of lipid peroxidation reactions, was measured using the MDA assay kit (Beijing Solarbio Science & Technology, Beijing, China) according to the manufacturer’s instructions [29]. Cells were plated in 6-well plates at a density of 1.0 × 10^5^ cells per well and cultured for 24 h to allow adherence, before exposure to 0, 4, and 8 μg/mL of GO-AgNPs for 24 h. Then, the cells were washed with PBS twice and MDA activities were quantitated by reading optical densities at 532 nm using a Synergy 2 multi-mode microplate reader (BioTek, USA).

### 2.10. Measurement of Lactate Dehydrogenase (LDH) Production

CFFCs were seeded in a 24-well culture plate and treated with 0, 4, and 8 μg/mL of GO-AgNPs for 24 h. LDH levels of cells in the culture medium were quantified using the LDH-cytotoxicity assay kit (Beijing Solarbio Science & Technology, Beijing, China) [29]. LDH activities were quantitated by reading optical densities at 490 nm using a Synergy 2 multi-mode microplate reader (BioTek, USA).

### 2.11. Measurement of Caspase-3 Activity

Caspase-3 activity was measured using a caspase-3 activity kit (Beijing Solarbio Science & Technology, Beijing, China) according to manufacturer’s instructions. Briefly, CFFCs were seeded in a 24-well culture plate and treated with 0, 4, and 8 μg/mL of GO-AgNPs for 24 h. Then, the cells were washed twice in PBS, lysed using lysis buffer, and centrifuged at 16,000× *g* at 4 °C for 10 min, and the supernatant was incubated with 10 µL of caspase-3 substrate for 7 h at 37 °C. Substrate cleavage was measured at 405 nm using a Synergy 2 multi-mode microplate reader (BioTek, USA).

### 2.12. Determination of Global 5-mC

Genomic DNA from cultured cells was purified with the DNeasy blood and tissue kit (Qiagen, Inc, Hilden, Germany). Global DNA methylation was determined according to the Methyl Flash Methylated DNA Quantification Kit (Colorimetric; Epigentek Group Inc., New York, NY, USA). Briefly, the percentage of 5-mC in 100 ng of DNA was proportional to the OD intensity in an ELISA plate reader at 450 nm. DNA methylation was calculated using the formula [((Sample OD – M3OD)/S)/((M4OD – M3OD) × 2)/P] × 100, where OD is the optical density; M3 is the negative control, an unmethylated polynucleotide containing 50% of cytosine; S is the amount of input sample DNA in nanograms; M4 is the positive control, a methylated polynucleotide containing 50% of 5-methylcystosine; and P is the amount of input positive control in nanograms. The relative amount of methylated DNA was expressed as a percentage of total DNA.

### 2.13. Quantitative Reverse Transcription PCR (RT-qPCR) Analysis

Total RNA was extracted from CFFCs using an RNA Isolation Kit (Thermo Scientific, Waltham, MA, USA) according to the manufacturer’s instructions. RNA samples were stored at −80 °C until use. The mRNA samples were reverse-transcribed into first-strand cDNA using the iScript cDNA Synthesis Kit (Bio-Rad Laboratories, Hercules, CA, USA) according to the manufacturer’s instructions. Quantitative analysis of the cDNA samples was performed using a CFX96 instrument (Bio-Rad Laboratories), using SYBR Green (Vazyme). Primers were designed based on the mRNA sequences of selected genes available in GenBank (Table 1). The PCR cycle was as follows: initial denaturation at 95 °C for 30 s, followed by 41 cycles of denaturation at 95 °C for 15 s, annealing at 60 °C for 15 s, and extension at 72 °C for 30 s. RT-qPCR was performed independently four times. The target genes were quantified by the delta-delta Ct method using CFX manager V1.1 software (Bio-Rad Laboratories). Normalization was performed using β-actin as the reference gene.

### 2.14. Statistical Analysis

The assessors were blinded to any stage of the methodological process. All results were expressed as the mean ± SD and analyzed using Origin 8.0 and SPSS 18.0 (IBM Corp., Armonk, NY, USA). The statistical significance of the changes between tested groups and the control group was analyzed by one-way ANOVA followed by Dunnett’s multiple comparison. The level of statistical significance was set at *p* < 0.05. All experiments were performed at least three times.

## 3. Results

### 3.1. Characterization of GO-AgNPs

**Figure 1:**
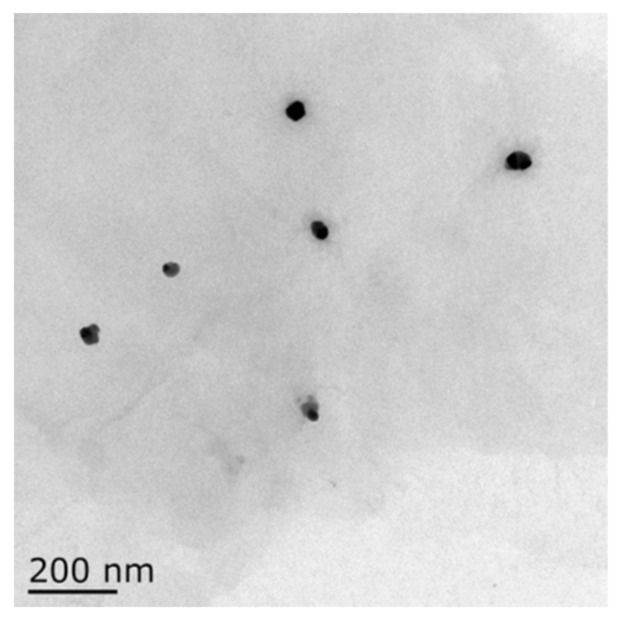
Size determination analysis of graphene oxide–silver nanoparticle (GO-AgNP) nanocomposites by transmission electron microscopy (TEM). The TEM image showed that the size of GO-AgNPs was about 20 nm.

TEM analysis was conducted to confirm the structural and surface morphology of the GO-AgNP nanocomposite. The size distribution of the AgNPs was about 20 nm, as shown in the image of TEM (Figure 1). GO-AgNPs images clearly showed transparent, single-layer sheets containing flake-like wrinkles in which AgNPs were homogeneously arranged on the micron scale of the GO sheets, which presented no evidence of agglomeration.

### 3.2. Effect of GO-AgNPs on Caprine Fetal Fibroblast Cell (CFFC) Viability

**Figure 2:**
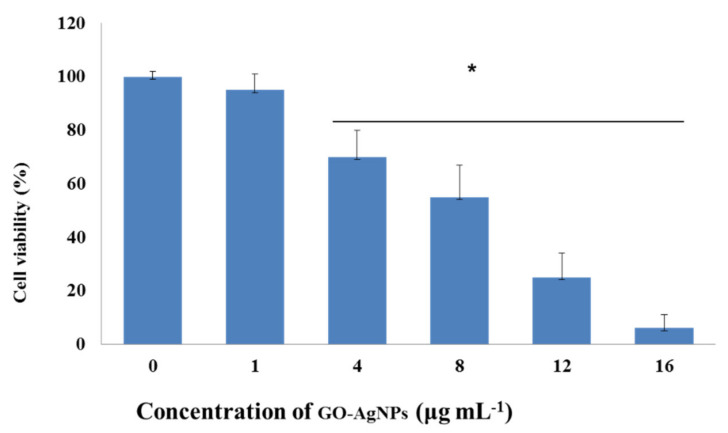
Effects of GO-AgNPs on the proliferation of caprine fetal fibroblast cells (CFFCs). CFFCs were exposed to 0, 1, 4, 8, 12, and 16 µg/mL of GO-AgNPs for 24 h. The percentage of cell viability was then calculated relative to the control group (0 µg/mL). Values are presented as the mean ± SD of four independent experiments (* *p* < 0.05).

For assessment of the potential cytotoxic effect of GO-AgNPs on CFFCs, cell viability following GO-AgNPs treatment was determined using the CCK-8 assay. As shown in Figure 2, there were no significant differences in cell viability between control cells and those exposed to 1 µg/mL of GO-AgNPs for 24 h; however, the viability of cells was significantly reduced when the concentration increased (4, 8, 12, and 16 µg/mL), suggesting that GO-AgNPs induce toxicity in CFFCs in a dose-dependent manner.

### 3.3. Effect of GO-AgNPs on Cell Morphology

**Figure 3:**
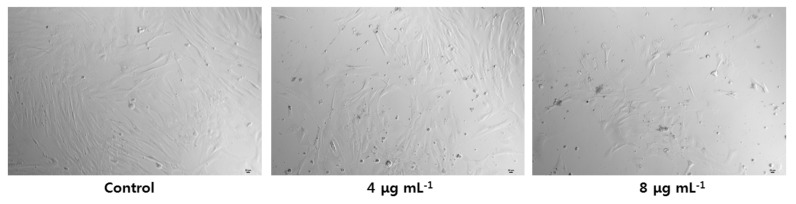
Cell morphology following treatment with GO-AgNPs. Caprine fetal fibroblast cells (CFFCs) were treated with 0, 4, and 8 µg/mL of GO-AgNPs for 24 h and then visualized under a phase-contrast microscope (magnification, 100×). Scale bar = 20 μm.

The morphologies of CFFCs after exposure to GO-AgNPs for 24 h are shown in Figure 3. Cell morphology of the control group was uniform with spindle-shaped cells. CFFCs that had been exposed to 4 and 8 µg/mL of GO-AgNPs exhibited marked morphological changes and showed cell membrane breakage, with obvious reduction in the number of cells in the group exposed to 8 µg/mL of GO-AgNPs.

### 3.4. Effect of GO-AgNPs on Reactive Oxygen Species (ROS) Production

**Figure 4:**
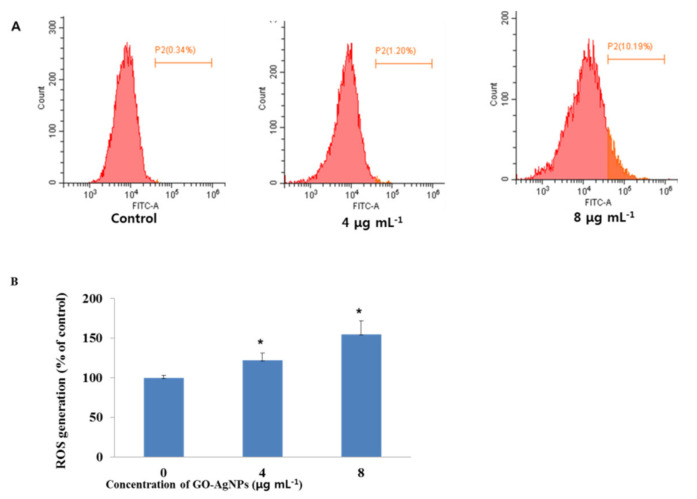
Total reactive oxygen species (ROS) generation in GO-AgNP-treated cells. Caprine fetal fibroblast cells (CFFCs) were treated with 0, 4, and 8 µg/mL of GO-AgNPs for 24 h and analyzed by FACS (**A**). The percentage of ROS generation relative to the untreated control group (0 µg/mL) (**B**). Values are presented as the mean ± SD of four independent experiments (* *p* < 0.05).

To study whether GO-AgNPs induce an oxidative impact involving apoptosis, the intracellular ROS level in CFFCs was analyzed. As shown in Figure 4, the level of intracellular ROS in CFFCs significantly increased (*p* < 0.05) when the cells were treated with 4 and 8 µg/mL of GO-AgNPs for 24 h compared to the control group.

### 3.5. Effects of GO-AgNPs on Apoptosis

**Figure 5:**
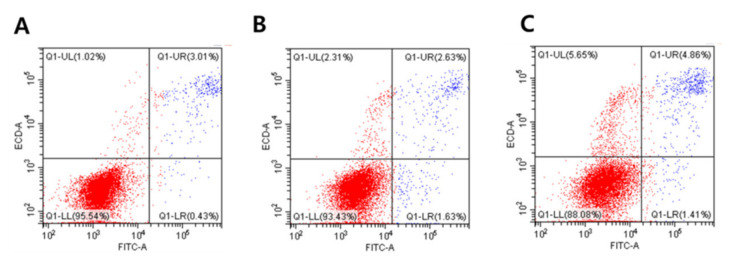

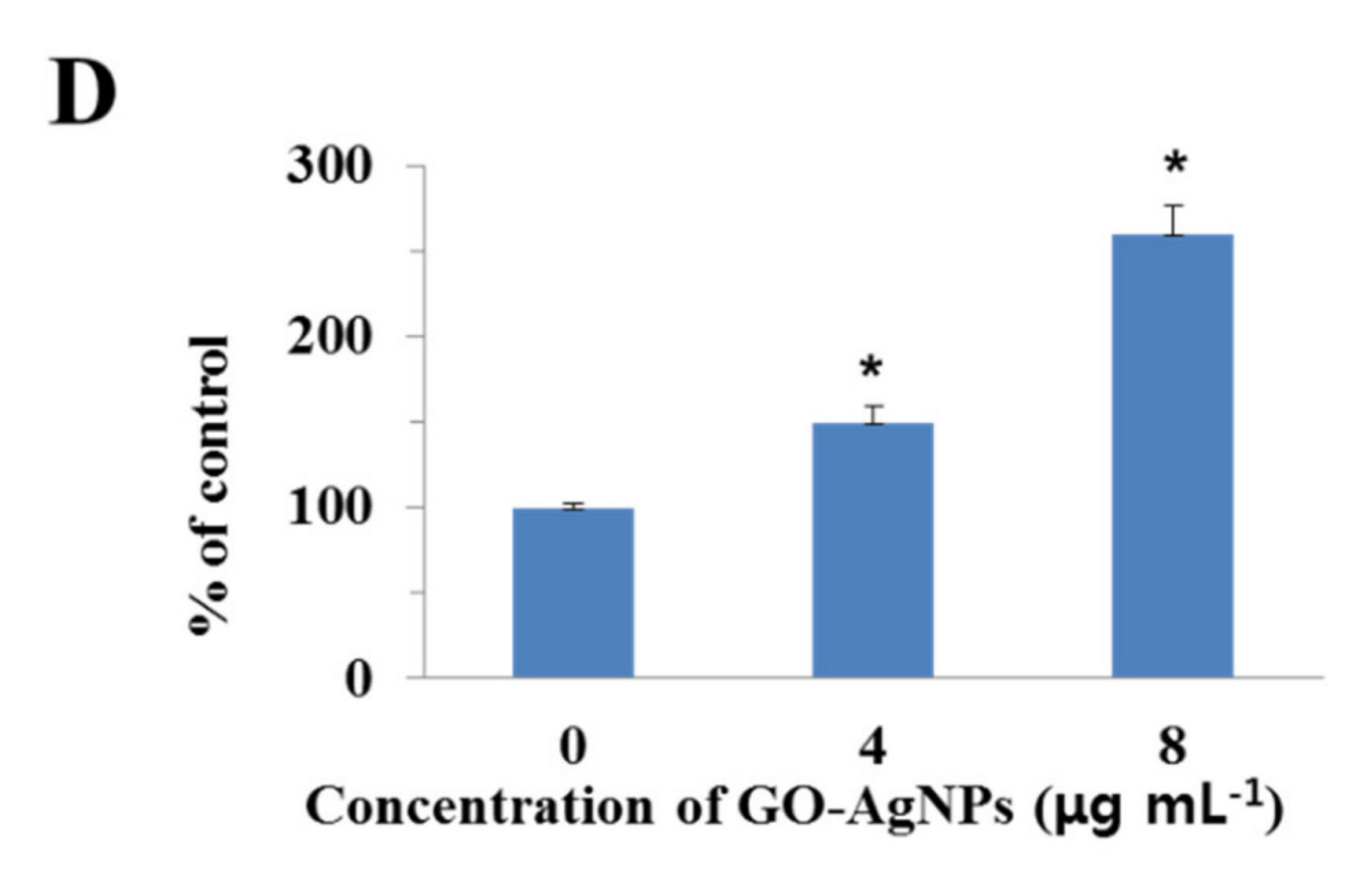
Evaluation of GO-AgNP-induced apoptotic cell death with the annexin V–FITC/propidium iodide (PI) staining assay. Caprine fetal fibroblast cells (CFFCs) were treated with 0 (**A**), 4 (**B**), and 8 µg/mL (**C**) of GO-AgNPs for 24 h, and FACS was carried out for detection of fractions of early apoptotic, late apoptotic, and necrotic CFFCs. The corresponding linear diagram of flow cytometry is shown in (**D**). Values are presented as the mean ± SD of five independent experiments (* *p* < 0.05).

The effect of GO-AgNPs on cell apoptosis was tested. An annexin V/PI apoptosis kit was used to quantify, by flow cytometry, the percentage of CFFCs undergoing apoptosis and dying. The results suggested that GO-AgNPs induce significant apoptosis and cell death in CFFCs (Figure 5).

### 3.6. Effects of GO-AgNPs on SOD Production

**Figure 6:**
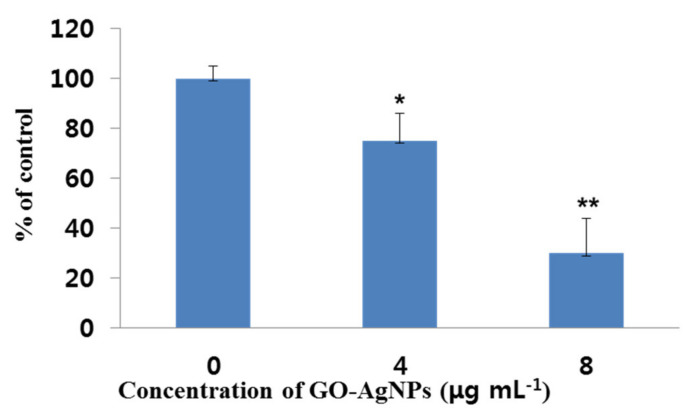
Measurement of superoxide dismutase (SOD) production in GO-AgNP-treated cells. Caprine fetal fibroblast cells (CFFCs) were treated with 0, 4, and 8 µg/mL of GO-AgNPs for 24 h. The percentage of SOD relative to the control group (0 µg/mL) was determined. Values are presented as the mean ± SD of four independent experiments (* *p* < 0.05; ** *p* < 0.01).

Effects of GO-AgNPs on the production of the anti-oxidant indicator SOD in CFFCs were determined with an SOD assay kit. As shown in Figure 6, the SOD activity decreased significantly (*p* < 0.05) in CFFCs treated with 4 µg/mL of GO-AgNPs for 24 h compared to the control group. Furthermore, CFFCs treated with 8 µg/mL of GO-AgNPs significantly decreased (*p* < 0.01) SOD activity compared to untreated CFFCs.

### 3.7. Effects of GO-AgNPs on MDA Production

**Figure 7:**
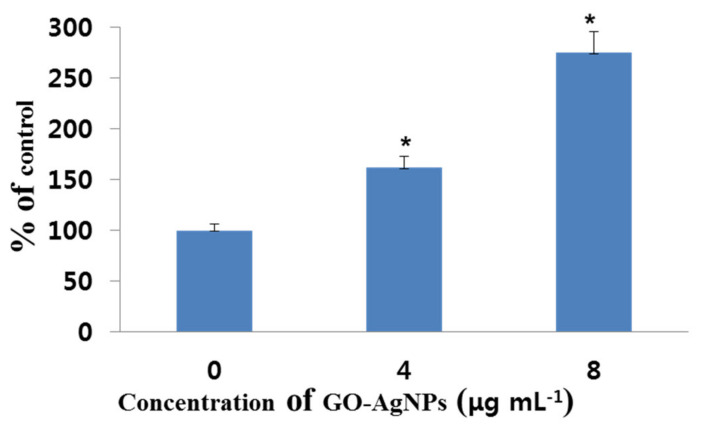
Measurement of malondialdehyde (MDA) production in GO-AgNP-treated cells. Caprine fetal fibroblast cells (CFFCs) were treated with 0, 4, and 8 µg/mL of GO-AgNPs for 24 h. The percentage of MDA relative to the control group (0 µg/mL) was determined. Values are presented as the mean ± SD of four independent experiments (* *p* < 0.05).

The production of MDA in CFFCs was determined using the MDA assay kit after treatment with 0, 4, and 8 µg/mL of GO-AgNPs for 24 h. The results showed that the levels of the oxidative damage indicator MDA increased significantly (*p* < 0.05) in the 4 and 8 µg/mL groups compared to the control group (Figure 7).

### 3.8. Effects of GO-AgNPs on LDH

**Figure 8:**
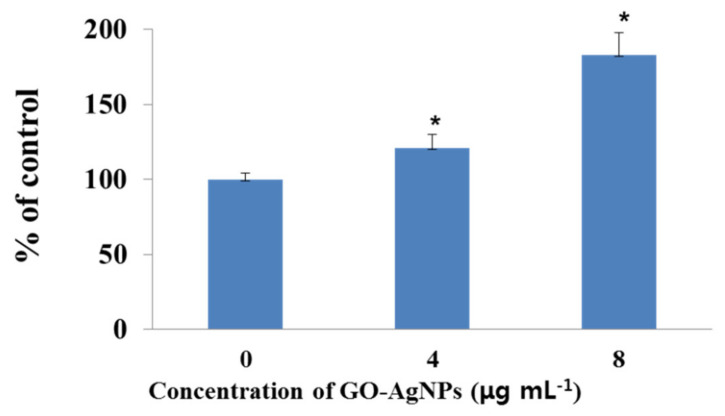
Measurement of lactate dehydrogenase (LDH) activity in GO-AgNP-treated cells. Caprine fetal fibroblast cells (CFFCs) were treated with 0, 4, and 8 µg/mL of GO-AgNPs for 24 h. The percentage of LDH activity relative to the control group (0 µg/mL) was determined. Values are presented as the mean ± SD of five independent experiments (* *p* < 0.05).

CFFCs were treated with 0, 4, and 8 µg/mL of GO-AgNPs for 24 h, and the level of leakage of LDH was measured. The results indicated that GO-AgNPs significantly increased the leakage level of LDH in CFFCs compared to the control group (Figure 8; *p* < 0.05).

### 3.9. Effects of GO-AgNPs on the Caspase-3 Activity

**Figure 9:**
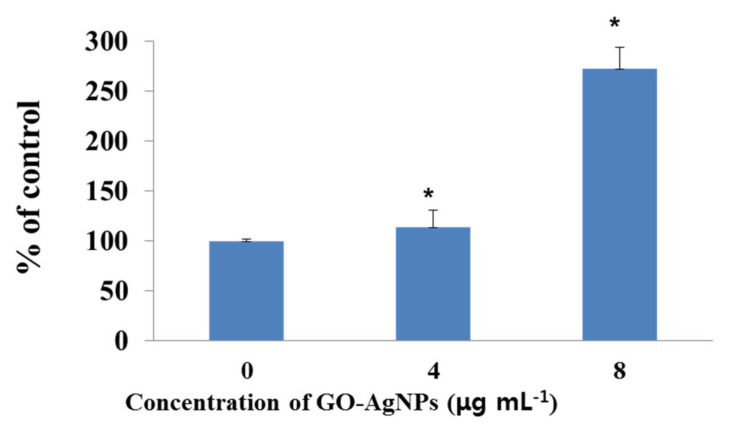
Measurement of caspase-3 activity in GO-AgNP-treated cells. Caprine fetal fibroblast cells (CFFCs) were treated with 0, 4, and 8 µg/mL of GO-AgNPs for 24 h. The percentage of caspase-3 activity relative to the control group (0 µg/mL) was determined. Values are presented as the mean ± SD of four independent experiments (* *p* < 0.05).

To confirm whether caspase-3 is involved in the apoptosis of CFFCs treated with different concentrations of GO-AgNPs (4 and 8 µg/mL), caspase-3 activity was measured by a caspase-3 kit. The activity of caspase-3 in the 4 and 8 µg/mL groups was significantly (*p* < 0.05) higher after treatment than that in the control group (Figure 9).

### 3.10. Effects of GO-AgNPs on Gene Expression

**Figure 10:**
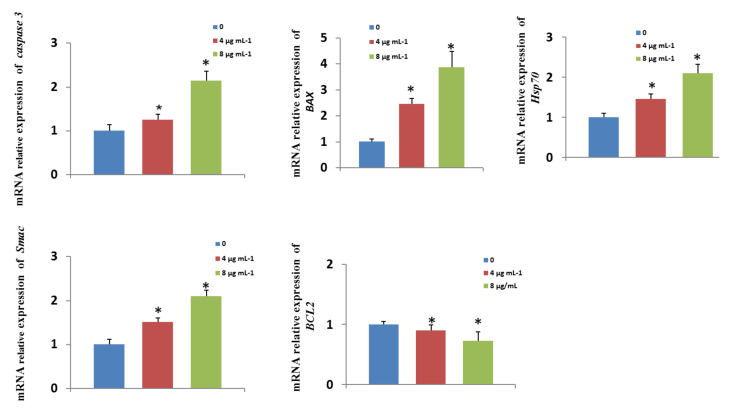
Effects of GO-AgNPs on apoptotic gene expression levels. Caprine fetal fibroblast cells (CFFCs) were treated with 0, 4, and 8 µg/mL of GO-AgNPs for 24 h. Relative mRNA levels of genes related to apoptosis were determined. Values are presented as the mean ± SD of four independent experiments (* *p* < 0.05).

To elucidate the possible molecular mechanisms underlying the negative effect of GO-AgNPs, the mRNA levels of pro- and anti-apoptotic genes as well as cell-death- and survival-related genes, including *caspase-3*, *BAX*, *Smac*, *Hsp70,* and *BCL2*, were measured in CFFCs treated with GO-AgNPs (0, 4, and 8 µg/mL) for 24 h. The results showed that the level of *caspase-3*, *BAX*, *Smac,* and *Hsp70* were significantly (*p* < 0.05) upregulated in GO-AgNP-treated cells compared to control cells (Figure 10). The level of the anti-apoptosis gene *BCL2* was significantly (*p* < 0.05) downregulated in GO-AgNP-treated cells compared to control cells (Figure 10).

### 3.11. Effects of GO-AgNPs on Global DNA Methylation

**Figure 11:**
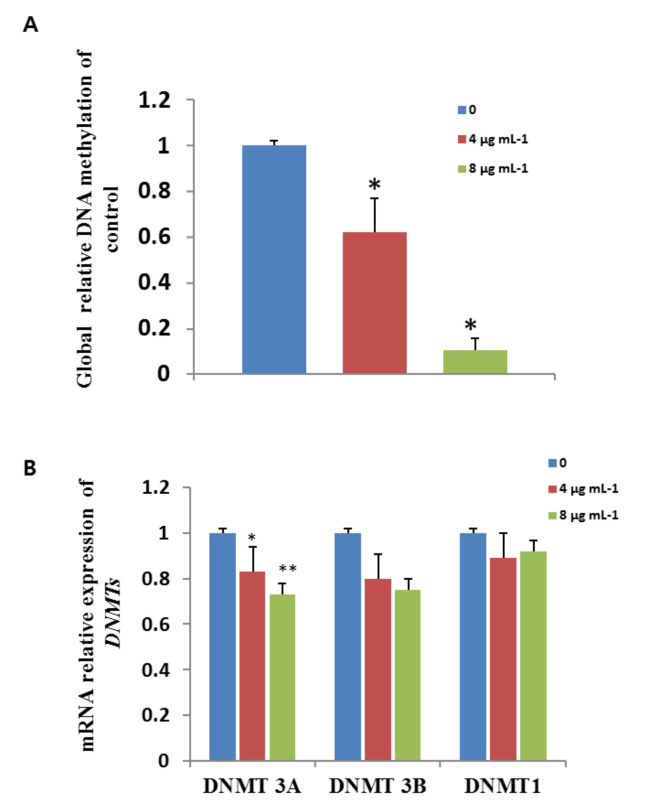
Effects of GO-AgNPs on global DNA methylation and gene expression levels. Caprine fetal fibroblast cells (CFFCs) were treated with 0, 4, and 8 µg/mL of GO-AgNPs for 24 h. Relative global DNA methylation (**A**) and mRNA levels of DNMTs (**B**) were determined. Values are presented as the mean ± SD of four independent experiments (* *p* < 0.05; ** *p* < 0.01).

Global DNA methylation levels decreased in CFFCs exposed to GO-AgNPs (4 and 8 µg/mL) compared to untreated CFFCs. The mean values of the 4 and 8 µg/mL GO-AgNP-treated CFFCs decreased to 62% and 10% (*p* < 0.05) of control cells, respectively (Figure 11A). As shown in Figure 11B, the mRNA expression levels of *Dnmt3A* significantly increased after exposure to GO-AgNPs (*p* < 0.05, *p* < 0.01). However, there was no significant difference in the expression of *Dnmt1* and *Dnmt3B* between GO-AgNP-treated groups and controls.

## 4. Discussion

Graphene-based nanomaterials have enormous applications in the field of nanomedicine due to their excellent biocompatibility and physicochemical properties [30]. As efficient support materials, graphene sheets can disperse and stabilize silver nanoparticles by preventing their agglomeration, which opens a way for the development of hybrid nanomaterials using both graphene and silver composites. Consequently, graphene-and-AgNP-based hybrid nanocomposites have been widely produced to evaluate their antibacterial and anticancer activity [31]. However, AgNPs can easily enter cells, thus affecting the physiology of organisms, which may show potential toxicity to both human and animal health or ecosystems [11]. Therefore, the adverse effects of GO-AgNP nanocomposites have been considered a major limitation for their broad applications. Numerous studies have proved the toxicological effects of GO-AgNP nanocomposites on normal animal and human cells [20,31,32]. However, the toxic effects of GO-AgNPs on the developing fetus (cells originating from the fetus) of livestock species have not been explored yet. In the present study, a GO-AgNP nanocomposite was synthesized using quercetin, and its surface and structural morphology as well as the uniform distribution of AgNPs on the GO sheets was confirmed using TEM. After that, the potential toxicity level of the synthesized GO-AgNPs on CFFCs was explored.

It has been reported that animals and human are frequently exposed to AgNPs via the routes of inhalation, dermal contact, and oral ingestion [11]. As an in vitro model, cell lines are frequently used for testing the toxic effects of different nanomaterials. For example, several studies have demonstrated that AgNPs induce toxicity via oxidative stress and apoptosis in mouse and rat cell lines [33,34]. AgNPs with a smaller particle size can easily enter and get distributed throughout cytoplasmic organelles [35]. However, smaller AgNPs (6 nm) are reported to be non-toxic to the mouse fibroblast line and the human keratinocyte cell line [36]. Similar results were reported that 5 µg/mL of rGO-Ag nanocomposite did not induce cytotoxicity in human normal cells (CHANG cells) but could slightly induce a toxic effect on HepG2 cells [32], which may ascribe the differences in toxicity mechanisms to the particular cell type [37]. The present data showed that 20 nm GO-AgNPs reduced cell growth and viability and induced morphological changes in a concentration-dependent manner. In our previous study, GO-AgNPs significantly decreased the human ovarian cancer cell viability with an IC50 of 5 µg/mL [26], which is lower than that in the present study, suggesting that CFFCs are less sensitive to GO-AgNPs than human cancer or mouse cells. AgNPs with different sizes and surface coatings or without coatings are likely to contribute to these different results. Lopes et al. [38] reported that coated AgNPs have a better dispersion ability and are exposed to cells in a better way than non-coated AgNPs. In addition, the toxicity level varies among the type and origin of the cell lines. For example, compared to L02 cells, HepG2 cells are more sensitive to AgNPs at the exposure level of 20-160 μg/mL [39].

One of the main mechanisms of toxicity induced by nanomaterials is that it causes oxidative stress through the generation of ROS and causes damage to cellular components, including DNA damage, abnormal activation of transcription factors, depletion of anti-oxidant molecules, binding and disabling of proteins, and damage to the cell membrane [11]. Oxidative stress inducing ROS is one of the proposed toxicological mechanisms of various nanomaterials such as Ag or Ag–graphene nanocomposites and can cause mitochondrial damage and initiation of lipid peroxidation [26,28,40]. Cytotoxicity of AgNPs is associated with increased production of ROS, which plays an important role in apoptosis induced by AgNPs [41]. Compared to pristine AgNPs, GO-AgNPs significantly induce the generation of ROS in the macrophages in a dose-dependent manner [42]. Especially, the generation of ROS and its association with oxidative stress in cells have been reported as critical indicators of graphene-based-nanomaterial-mediated toxicity, which causes DNA damage and reduced cell viability. A previous study investigated graphene-based-nanomaterial-mediated toxicity in biological systems as well as its response in various molecular pathways such as activating base excision repair and PI3K pathways in zebrafish larvae [43]. In the current study, GO-AgNPs treatment enhanced the generation of ROS by 1.4- and 1.8-fold in CFFCs treated with 4 and 8 µg/mL of GO-AgNPs for 24 h, respectively. Our results are consistent with previous reports on various cancer cell lines with graphene and graphene-related materials [16,26]. The upregulated ROS level in CFFCs alters mitochondrial functions and plays a key role in apoptosis induction, which was proved by the data of the annexin V/PI double-labeling assay and increasing levels of caspase-3. Present data suggest that the possible mechanisms of GO-AgNP-mediated toxicity in CFFCs include the stimulation of oxidative stress, which is responsible for upregulation of pro-apoptotic genes as well as downregulation of anti-apoptotic genes in CFFCs [16].

The increased levels of MDA and LDH are generally considered to imply cell injury. One of the adverse effects of oxidative stress is the lipid peroxidation of cell membranes. Many types of cells treated with AgNPs and GO have shown significantly increased levels of MDA, which is one of the final products of polyunsaturated fatty acid peroxidation in the cells [2,16,43,44,45]. Assessing the release of intracellular LDH in a cell, which results from the breakdown of and alteration in the permeability of the plasma membrane, is one of the markers for estimating cytotoxicity [16,40]. For instance, rGO-Ag increases LDH leakage in human cancer cells, thus resulting in cell death [16,26,46]. In the present study, the LDH level in the 4 µg/mL group was slightly higher than the control group, which is the same as the cell viability and apoptotic cell data. It means that although a low concentration of GO-AgNPs (4 µg/mL) seems to be toxic to cells, it may also result in some change in the cells. The present data indicated that the mechanism of increased levels of MDA and LDH in GO-AgNP-treated CFFCs may be due to ROS formation, which influenced the viability and proliferation of the cells, suggesting the possible cytotoxic effects of GO-AgNPs on CFFCs.

The apoptosis of cells is a highly conserved mechanism, and ROS is an important factor involved in the apoptotic process [47]. ROS induced by nanomaterials could result in nuclear DNA damage as well as leakage of lipids, proteins, and carbohydrates in the cell [34,45]. ROS production and lipid peroxidation induced by GO-AgNPs affect cellular redox homeostasis and decrease anti-oxidant levels [46]. It is well known that SOD plays an important role in anti-oxidant defense against oxidative stress in cells that can combat the accumulation of ROS and reduce oxidative injury. A decrease in SOD activity is an indicator of impairment of protective mechanisms and significantly contributes to cell damage [48]. It has been reported that AgNPs directly interact with SOD and CAT and altered the expression and activity of anti-oxidant enzymes (CAT, SOD, and GPX) [48]. The present data showed that the level of SOD significantly decreased in GO-AgNP-treated CFFCs. It suggests that GO-AgNPs decrease the levels of anti-oxidant molecules in the cells, which might be the reason for cytotoxicity.

Similarly, apoptotic and anti-apoptotic genes play an important role in cell survival and death. Several studies have reported oxidative stress and DNA damage as the mechanism for GO-AgNP-induced cytotoxicity and apoptosis of cancer cells [20]. A similar study reported that GO-AgNPs can cause oxidative damage and leakage of LDH and enhance the expression of apoptotic genes *p53*, *caspase-3*, *caspase-9*, *Bax*, and *c-myc*, thus leading to mitochondrial dysfunction and triggering apoptosis [29], and all apoptotic pathways appear to terminate in the activation of the caspase family of proteases [49]. Moreover, oxidative stress induced by GO-AgNPs is reported to increase the total expression of *Bax* in a dose-dependent manner and downregulate the expression of the anti-apoptotic gene *BCL-2* [46]. Heat shock protein 70 (HSP70) allows cells to adapt to gradual environment changes and is considered to play a crucial role in environmental stress tolerance [50]. The present data also showed that GO-AgNPs upregulate the expression of *HSP70* and pro-apoptotic genes such as *caspase-3*, *Bax*, *and Smac* and downregulate anti-apoptotic genes such as *Bcl-2*. Similarly, rGO-Ag was reported to cause dynamic balance troubles in the level of *Bcl-xl* and *Bcl-2*, and downregulation of *c-myc* triggers apoptosis along with *p53* [29], which may induce apoptosis of CFFCs.

It has been reported that nanomaterials induce epigenetic changes, including DNA methylation, histone modifications, and noncoding RNA-mediated regulation of gene expression [50]. Nanoparticle-mediated global DNA hypomethylation or hypermethylation can be corroborated with increased generation of ROS [14], which is now known to cause many human diseases, including cancer [51]. Nanomaterial-induced epigenetic changes are also shown to be cell type, time, and dose dependent. For example, ZnO-NPs induced increasing levels of ROS and significantly resulted in global reduction in 5-mC [52], while AgNP exposure to pregnant mice via intravenous infusion significantly altered the methylation levels of differentially methylated regions of *Zac1* and disrupted the imprinted gene expression [53]. However, exposure of AgNPs via the abdominal subcutaneous route had detrimental effects on spermatogenesis and the quality of sperm in neonatal mice [54]. The current research showed that GO-AgNP treatment causes a significant reduction in global 5-mC in CFFCs, which was further proved by the decreased expression of *DNMT3A*. After recovering from the treatment of AgNPs, HT22 mouse hippocampal neuronal cells showed increased levels of 5-mC, DNMT3A, and DNMT3B [54]. The different expressions of *DNMT3A* and *DNMT3B* in our study may be explained as *Dnmt3a* or *Dnmt3b* selectively recognizing heterochromatin [55]. Therefore, the global DNA hypomethylation in the GO-AgNP-treated CFFCs might be the result of aberrant oxidative stress.

## Figures and Tables

**Table 1:** Primers used for quantitative reverse transcription PCR analysis.

Gene	Primer sequence (5‘–3‘)	Product Size (bp)
*caspase-3*	F: CCATGGTGAAGAAGGAATCATTT R: TCCCCTCTGAAGAAACTTGCTAA	78
*BAX*	F: GCATCCACCAAGAAGCTGAG R: CCGCCACTCGGAAAAAGAC	120
*Smac*	F: TGTTCCAGTCGTGGCTAACTT R: AAAGACACAGCCCTCCTCATT	171
*BCL2*	F: ATGTGTGTGGAGAGCGTCA R: AGAGACAGCCAGGAGAAATC	113
*Hsp70*	F: TCAGGACTCAATCTGCATCG R: ATCCGCATTTCTGGTTATCA	210
*DNMT3A* *DNMT3B* *DNMT1* *β-actin*	F: CTTGGAGAAGCGGAGTGAGC R: GTGCAGCAGCCATTCTCTACAG F: AGCCCCTACCTCACCATC R: CTGATACTCGGTGCTGTCTGC F: GAGGAGGCTGCCAAGGACT R: CAAACACCGCATACGACACAC F: TCACGGAGCGTGGCTACAG R: CCTTGATGTCACGGACGATTT	138 156 134 127

Abbreviations: F, forward; R, reverse.

## Data Availability

Data available in a publicly accessible repository.
